# Obesity and smoking are factors associated with poor prognosis in patients with bacteraemia

**DOI:** 10.1186/1471-2334-7-13

**Published:** 2007-03-09

**Authors:** Reetta Huttunen, Janne Laine, Jukka Lumio, Risto Vuento, Jaana Syrjänen

**Affiliations:** 1 Department of Internal Medicine, Tampere University Hospital, PL 2000, FIN-33521 Tampere, Finland; 2 Medical School, University of Tampere, Teiskontie 35 (K-building), FIN-33521 Tampere, Finland; 3 Centre for Laboratory Medicine, Tampere University Hospital, Biokatu 4, PL 2000, FIN-33521 Tampere, Finland

## Abstract

**Background:**

Bacteraemia is still a major cause of case fatality in all age groups. Our aim was to identify the major underlying conditions constituting risk factors for case fatality in bacteraemia patients.

**Methods:**

The study involved 149 patients (79 male and 70 female) with bacteraemia caused by *Staphylococcus aureus (S. aureus) *(41 patients), *Streptococcus pneumoniae (Str. pneumoniae) *(42 patients), β-hemolytic streptococcae (β-hml str.) (23 patients) and *Eschericia coli (E. coli) *(43 patients). Underlying diseases, alcohol and tobacco consumption and body mass index (BMI) were registered. Laboratory findings and clinical data were registered on admission and 6 consecutive days and on day 10–14. Case fatality was studied within 30 days after positive blood culture. Associations between underlying conditions and case fatality were studied in univariate analysis and in a multivariate model.

**Results:**

Nineteen patients (12.8%) died of bacteraemia. We found obesity (p = 0.002, RR 9.8; 95% CI 2.3 to 41.3), smoking (p < 0.001, RR 16.9; 95% CI 2.1 to 133.5), alcohol abuse (p = 0.008, RR 3.9; 95% CI 1.3 to 11.28), COPD (p = 0.01, RR 8.4; 95% CI 1.9 to 37.1) and rheumatoid arthritis (p = 0.045, RR 5.9; 95% CI 1.2 to 28.8) to be significantly associated with case fatality in bacteraemia in univariate model. The median BMI was significantly higher among those who died compared to survivors (33 vs. 26, p = 0.003). Obesity and smoking also remained independent risk factors for case fatality when their effect was studied together in a multivariate model adjusted with the effect of alcohol abuse, age (continuos variable), sex and causative organism.

**Conclusion:**

Our results indicate that obesity and smoking are prominent risk factors for case fatality in bacteraemic patients. Identification of risk factors underlying fatal outcome in bacteraemia may allow targeting of preventive efforts to individuals likely to derive greatest potential benefit.

## Background

Bacteraemia is a common infection with significant morbidity and in most severe instances also mortality. The importance of underlying conditions such as immunosupression, ultimately and rapidly fatal diseases and chronic organ insufficiency for bacteraemia outcome has been emphasized in a number of studies [1-3].

Obesity is an increasing health concern in Western countries. In the general population, a relationship between obesity and increased mortality has been shown [4-6]. Data on the incidence and outcome of specific infections, especially community-acquired infections, in obese people are so far limited [7]. There are no papers on the effect of obesity on case fatality in bacteraemia patients.

Smokers and alcohol abusers display an increased susceptibility to bacterial infections, especially those involving the lung. In addition, smoking [8] and alcoholism [9] are major risk factors for invasive pneumococcal disease. There are data showing that alcoholism is a risk factor for case fatality in *S. aureus *[10] and *Str. pneumoniae *bacteraemia [11,12]. However, some studies have found no statistically significant association between alcoholism and case fatality [2,3,13]. A recent study of epidemiological and clinical features as potential prognostic factors for outcomes of hospital-acquired bacteraemia found no differences between non-survivors and survivors in sex, age or smoking habit [14]. In bacteraemias other than pneumococcal, the effect of smoking and alcoholism on case fatality has not been widely studied.

Moreover, studies of bacteraemia with varying disease severity are few in number. Most materials in this field consist of patients merely treated with severe infection in ICU; patients originally evincing milder signs and symptoms of infection are not included. The aim of our study was to elucidate the underlying conditions, especially the role of obesity, smoking and alcohol abuse, as risk factor for case fatality in bacteramia.

## Methods

The study material comprised 149 patients (79 male and 70 female) with bacteraemia hospitalized in Tampere University Hospital, Tampere, Finland, from June 1999 to February 2004. Their ages ranged from 16 to 93 years (mean 59 years).

In our hospital blood cultures are routinely taken from patients with symptoms or signs of systemic infection (fever or hypothermia, tachycardia or tachypnea combined with leucocytosis or leucopenia and/or elevated C-reactive protein (CRP)). The study focused on patients with bactaeremia caused by *S. aureus, Str. pneumoniae*, β-hml str. or *E. coli*, the most common causative organisms among community-acquired bacteraemia. Patients were identified according to microbiological culture finding. The clinicians (J.S. or J.La.) were informed by clinical microbiologist (R.V.) of a positive blood culture from Mondays to Thursdays and the patients were enrolled to the study whenever possible to adjust to the daily schedule. We were able to recruit zero to two patients per week during the study period. Since the clinicians were unable to know any details about the patients nor their disease severity before the recruitment, the selection was purely based on the blood culture finding. After being informed by clinical microbiologist the clinician asked patients to participate and interviewed and examined those consenting. Information was gathered from hospital records at the time of hospital visit and hospital records were also reviewed after hospitalization (R.H.). Altogether 149 out of 152 patients agreed to participate. The causative organisms involved were *S. aureus *(41 patients, 27.5 %), *Str. pneumoniae *(42 patients, 28.2%), *β-hml str*. (23 patients, 15.4%), and *E. coli *(43 patients, 28.9%). Patients with two episodes of bacteraemia were not included in the study. During the period in question the BACTEC 9240 (BD Diagnostic Systems, Sparks, MD, USA) blood culture system with standard media was used. Bacteraemia was defined hospital-aqcuired (nosocomial) if positive blood culture was drawn >48 h after admission.

Underlying conditions, social status and alcohol and tobacco consumption were registered. Alcohol abuse was defined as consumption of 300 g absolute alcohol per week or a known social or medical problem due to alcohol use. Smoking habits were registered and patients were defined as current smokers, ex-smokers i.e. those who have stopped smoking and nonsmokers i.e. those who have never smoked (data available from 136 out of 149 patients). Calculation of BMI was based on weight and length as reported by the patient on admission. BMI was recorded and patients were defined as obese if their BMI was > 30 (data available from 114 out of 149 patients; 10/19 of deceased patients and 104/128 of survivors). Chronic diseases were registered and McCabe (and Jackson) classification was used to assess the severity of the underlying medical condition [15]. Preceding corticosteroid treatment was registered if corticosteroids were used in a dose of over 5 mg per day during 1 month before the bacteremia episode. All patients were treated with an empiric antibiotic regimen and antimicrobial treatment was changed according to culture results if necessary.

Patients were closely monitored during hospitalization and severely ill subjects were transferred to the ICU. Clinical data were registered on admission and during 6 consecutive days and on day 10–14 after admission. Possible need for mechanical ventilation was registered. Alterations in mental status were evaluated on the Glasgow Coma Scale (GCS). A patient was classified hypotensive if mean arterial pressure (MAP) was < 70 mmHg during the bacteraemia episode.

A SOFA score (Sequential Organ Failure Assessment) [16] was calculated on 1–3 days after positive blood culture finding (data available from 135/149 patients). Severe organ failure was documented if the SOFA score was ≥ 4. A patient did not need ICU treatment if the SOFA score was lower than 4. Laboratory findings were registered on admission, during 6 consecutive days and on day 10–14. The laboratory tests included plasma C-reactive protein (mg/l), blood white cell count (× 10^9^/l), blood platelets (× 10^9^/l), plasma bilirubin (μmol/l), plasma creatinine level (μmol/l), plasma alanine aminotransferase (U/l) and plasma alkaline phosphatase (U/l). The case fatality rate was studied within 30 days after the positive blood culture (day 30 case fatality).

The study was approved by the Ethics Committee of Tampere University Hospital. Written informed consent was obtained from patients or first degree relatives.

The SPSS package (version 7.5) was used for statistical analyses and a two-sided p-value < 0.05 was taken as the level for statistical significance. Categorical data were analysed by *X*^*2 *^test or Fisher's exact test when appropriate. Nonparametric data were analysed by Mann-Whitney U-test. Multivariate logistic regression analysis (method enter) was used to assess the effects of independent factors on mortality, controlling for differences in other factors possibly affecting the outcome. Risk ratios (RRs) were expressed with their 95% confidence intervals (CI). We studied the effect of obesity, smoking and alcohol abuse in a multivariate model adjusted with the effect of age (continuous variable), sex and organism (*S. Aureus, Str. Pneumoniae*, β-hl str., or *E. coli*).

## Results

Nineteen patients (12.8%) died of bacteraemia. The case fatality rate differed in bacteraemias caused by different organisms: *S. aureus *8/41 (19.5%), *Str. pneumoniae *8/42 (19.0%), β-hml str.2/23 (8.7%), and *E. coli *1/43 (2.3%). Bacteraemia was community-acquired in 119 patients (79.9%) and nosocomial in 30 (20.1%).

Predisposing factors and underlying diseases of bacteraemia are given in Table 1. Four differed bacteraemias differed statistically significantly from each other in the number of smoking patients (current smokers or ex-smokers, p = 0.007). Smoking was common in pneumococcal bacteraemic patients (65.0% of patients current smokers or ex-smokers) whereas 27.5% of *E. coli *bacteraemic patients were current smokers or ex-smokers. *S. aureus *bacteraemia was the most common pathogen in patients with rheumatoid arthritis. Bacteramias differed statistically significantly from each other in the number of male patients (p = 0.003). Only 30.2% of *E. coli *bacteramia patients were male whereas 68.3% of *S. aureus *bacteraemic patients were male. *S. aureus *was a common pathogen in nosocomial infection; 39% of *S. aureus *bacteraemias were nosocomial infections.

**Table 1:** Predisposing factors and underlying diseases of bacteraemia

Predisposing factor	All patients	*S. aureus*	*Str. pneumoniae*	β-haemolytic streptococci	*E. coli*	p-value
	N = 149	N = 41	N = 42	N = 23	N = 43	
	n (%)	n (%)	n (%)	n (%)	n (%)	
Obesity^a^	27 (23.7)	9 (25.7)	4 (16.7)	6 (31.6)	8 (22.2)	0.698
Current smoker or ex-smoker^b^	66 (48.5)	18 (48.6)	26 (65.0)	11 (57.9)	11 (27.5)	0.007
Alcohol abuse	24 (16.1)	5 (12.2)	9 (21.4)	6 (26.1)	4 (9.3)	0.211
Type 1 diabetes	5 (3.4)	1 (2.4)	-	-	4 (9.3)	0.1
Type 2 diabetes	29 (19.5)	9 (22.0)	5 (11.9)	4 (17.4)	11 (25.6)	0.426
COPD	8 (5.4)	-	5 (11.9)	2 (8.7)	1 (2.3)	0.053
Haematological malignancy	11 (7.4)	2 (4.9)	3 (7.1)	1 (4.3)	5 (11.6)	0.723
Solid malignancy	15 (10.1)	6 (14.6)	3 (7.1)	1 (4.3)	5 (11.6)	0.589
Rheumatoid arthritis	7 (4.7)	5 (12.2)	-	1 (4.3)	1 (2.3)	0.034
Liver cirrhosis	4 (2.7)	2 (4.9)	-	1 (4.3)	1 (2.3)	0.511
Cardiac disease^c^	44 (29.5)	14 (34.1)	10 (23.8)	6 (26.1)	14 (32.6)	0.705
McCabe II or III^d^	24 (16.1)	8 (19.5)	5 (11.9)	2 (8.7)	9 (20.9)	0.462
Corticosteroid treatment^e^	18 (12.1)	6 (14.6)	4 (9.5)	2 (8.7)	6 (14.0)	0.857
Chronic disease^f^	117 (78.5)	33 (80.5)	27 (64.3)	17 (73.9)	40 (93.0)	0.013
Age >60 years	78 (52.3)	21 (51.2)	20 (47.6)	10 (43.5)	27 (62.8)	0.391
Male sex	79 (53.0)	28 (68.3)	24 (57.1)	14 (60.9)	13 (30.2)	0.003
Nosocomial infection	30 (20.1)	16 (39.0)	4 (9.5)	3 (13.0)	7 (16.3)	0.004

^a^(BMI >30), data available from 114 patients

^b^Data available from 136 patients

^c^Coronary artery disease, valvular disease or documented heart failure

^d^McCabe class II or III: ultimately fatal or rapidly fatal disease

^e^Corticosteroids used in a dose of over 5 mg per day during one month prior to the episode of bacteraemia

^f^At least one chronic disease

The sources of the infection were identifiable in 132 patients and are listed in Table 2. Clinical data on all patients and on patients with bacteraemia caused by different organisms are shown in Table 3. The case fatality rate in relation to predisposing factors and underlying diseases is given in Table 4. Obesity, smoking, alcohol abuse, COPD and rheumatoid arthritis proved significant risk factors for case fatality in univariate analysis (table 4).

**Table 2:** Source of bacteraemia^a^

Focus	All	*S. aureus*	*Str. pneumoniae*	β-hml str.	*E. coli*
	n = 170^b^	n = 56^b^	n = 45^b^	n = 29^b^	n = 40^b^
	(%)	(%)	(%)	(%)	(%)
Pneumonia	39 (22.9)	2 (3.6)	35 (77.8)	2 (6.9)	0 (0)
Skin	37 (21.8)	19 (33.9)	2 (4.4)	16 (55.2)	0 (0)
Urinary	30 (17.6)	1 (1.8)	0 (0)	1 (3.4)	28 (70.0)
Source unknown	17 (10.0)	3 (5.4)	4 (8.9)	3 (10.3)	7 (17.5)
Osteomyelitis/spondylitis	15 (8.8)	12 (21.4)	0 (0)	3 (10.3)	0 (0)
Gall bladder	7 (4.1)	0 (0)	3 (6.7)	0 (0)	4 (10.0)
Endocarditis	6 (3.5)	6 (10.7)	0 (0)	0 (0)	0 (0)
Arthritis	6 (3.5)	5 (8.9)	0 (0)	1 (3.4)	0 (0)
Mediastinitis	4 (2.4)	4 (7.1)	0 (0)	0 (0)	0 (0)
Meningitis	3 (1.8)	1 (1.8)	1 (2.2)	1 (3.4)	0 (0)
Gynaecological	3 (1.8)	0 (0)	0 (0)	2 (6.9)	1 (2.5)
Intravenous/intra-arterial catheter-related	3 (1.8)	3 (5.4)	0 (0)	0 (0)	0 (0)

^a^One patient may have several focuses

^b^Indicating the number of focuses

**Table 3:** Clinical data on 149 patients with 4 different bacteraemias

Disease severity	All	*S. aureus*	*Str. Pneumoniae*	β-hemolytic streptococci	*E. coli*	p-value*
	n = 149	n = 41	n = 42	n = 23	n = 43	
	(%)	(%)	(%)	(%)	(%)	
Died^a^	19 (12.8)	8 (19.5)	8 (19.0)	2 (8.7)	1 (2.3)	0.031
Needed ICU stay	47 (31.5)	15 (36.6)	16 (38.1)	10 (43.5)	6 (14.0)	0.029
Needed mechanical ventilation	22 (14.8)	7 (17.1)	10 (23.8)	3 (13.0)	2 (4.7)	0.092
Needed CVVHD	7 (4.7)	5 (12.2)	0 (0)	2 (8.7)	0 (0)	0.006
Needed haemodialysis	2 (1.3)	2 (4.9)	0 (0)	0 (0)	0 (0)	0.097
Sofa Score >4^b^	39 (28.9)	9 (25.0)	10 (26.3)	11 (50.0)	9 (23.1)	0.122
Low platelet count^c^	21 (14.1)	8 (19.5)	3 (7.1)	4 (17.4)	6 (14.0)	0.411
Elevated bilirubin level^d^	51 (34.2)	14 (34.1)	12 (28.6)	10 (43.5)	15 (34.9)	0.687
Lowered GCS^e^	60 (40.3)	19 (46.3)	18 (42.9)	10 (43.9)	13 (30.2)	0.45
Hypotensive^f^	56 (37.6)	13 (31.7)	19 (45.2)	11 (47.8)	13 (30.2)	0.299

^a^Death due to bacteraemia episode occurred within 30 days from the day of positive blood culture.

^b^SOFA score was registered 1–4 days after positive blood culture finding (median 3 days). Data available from 135 patients.

^c^Platelet count at least once lower than 50 10E^9^/L 0–8 days after positive blood culture

^d^Bilirubin level elevated (>25 μmol/L) at least once 0–8 days after positive blood culture

^e^Glasgow coma scale lowered (<15) at least once 0–6 days after positive blood culture

^f^Hypotensive (MAP <70 mmhg) at least once 0–6 days after positive blood culture

*The difference between groups of patients with bacteraemia caused by different organisms

**Table 4:** Case fatality in relation to predisposing factors and underlying diseases (univariate analysis)

Predisposing factor	All patients	Deceased	Survivors	p-value	RR (95% CI)
	N = 149	N = 19	N = 130		
	n (%)	n (%)	n (%)		
Obesity^a^	27 (23.7)	7 (70.0)	20 (19.2)	*0.002*	*9.8 (2.3–41.3)*
Current smoker or ex-smoker^b^	66 (48.5)	13 (92.9)	53 (43.4)	*<0.001*	*16.9 (2.1–133.5)*
Alcohol abuse	24 (16.1)	7 (36.8)	17 (13.1)	*0.008*	*3.9 (1.3–11.2)*
Type 1 diabetes	5 (3.4)	0 (0)	5 (3.8)	*1*	*1.0 (0.9–1.0)*
Type 2 diabetes	29 (19.5)	6 (31.6)	23 (17.7)	*0.153*	*2.1 (0.7–6.2)*
COPD	8 (5.4)	4 (21.1)	4 (3.1)	*0.01*	*8.4 (1.9–37.1)*
Haematological malignancy	11 (7.4)	0 (0)	11 (8.5)	*0.36*	*0.9 (0.9–1.0)*
Solid malignancy	15 (10.1)	3 (15.8)	12 (9.2)	*0.41*	*1.8 (0.5–7.2)*
Rheumatoid arthritis	7 (4.7)	3 (15.8)	4 (3.1)	*0.045*	*5.9 (1.2–28.8)*
Liver cirrhosis	4 (2.7)	1 (5.3)	3 (2.3)	*0.42*	*2.4 (0.2–23.8)*
Cardiac disease^c^	44 (29.5)	6 (31.6)	38 (29.2)	*0.834*	*1.1 (0.4–3.2)*
McCabe II or III^d^	24 (16.1)	3 (15.8)	21 (16.2)	*1*	*1.0 (0.3–3.6)*
Corticosteroid treatment^e^	18 (12.1)	2 (10.5)	16 (12.3)	*0.824*	*0.8 (0.2–4.0)*
Chronic disease^f^	117 (78.5)	18 (94.7)	99 (76.2)	*0.065*	*5.6 (0.7–43.9)*
Age over 60 years	78 (52.3)	11 (57.9)	67 (51.5)	*0.604*	*1.3 (0.5–3.4)*
Male sex	79 (53.0)	14 (73.7)	65 (50.0)	*0.053*	*2.8 (1.0–8.2)*
Nosocomial infection	30 (20.1)	1 (5.3)	29 (22.3)	*0.084*	*0.2 (0.03–1.5)*

^a^(BMI >30), data available from 114 patients

^b^Data available from 136 patients

^c^Coronary artery disease, valvular disease or documented heart failure

^d^McCabe class II or III: ultimately fatal or rapidly fatal disease

^e^Corticosteroids used in a dose of over 5 mg per day during one month prior to the episode of bacteraemia

^f^At least one chronic disease

Myocardial infarction occurred in 11 (7.4%) patients and cerebral infarction in 7 (4.7%) during the month following positive blood culture. Twenty-six per cent of patients older than 60 years were treated in ICU compared to 38 per cent of those aged 60 or younger (p = 0.104).

### Obesity

Day 30 case fatality was higher in obese bacteraemic patients than in nonobese patients (25.9% vs. 3.4%, p = 0.002, RR 9.8; 95% CI 2.3–41.3). The median BMI was significantly higher among those who died compared to survivors (33 vs. 26, p = 0.003). Obese and nonobese study groups did not differ statistically significantly from each other in numbers needing ICU treatment (25.9% vs. 28.7%, p = 0.78, RR 0.9; 95% CI 0.3–2.3). Obese patients needed mechanical ventilation more often than nonobese, but the difference was not statistically significant (18.5% vs 8.0%, p = 0.152, RR 2.6; 95% CI 0.8–9.0). However, more obese than nonobese patients died in ICU treatment (5/7 vs. 3/25; p = 0.005, RR 18.3; 95% CI 2.4–140.4). The obese had high SOFA scores (value >4 on day 1–3 after positive blood culture finding) more often than nonobese, but the difference was not statistically significant (44.0% vs 23.8%, p = 0.05, RR 2.5; 95% CI 1.0–6.5). The obese and nonobese groups did not differ statistically significantly in the occurrence of hypotension (37.0% vs 31.0%, p = 0.561, RR 1.3; 95% CI 0.5–3.2), or in the number of patients with neurological deficit (lowered GCS) (44.4% vs 33.3%, p = 0.293, RR 1.6; 95% CI 0.7–3.9)

Forty-four per cent of obese bacteraemic patients had previously been diagnosed with type 2 diabetes as against 12.6% of nonobese patients (p < 0.001). Patients with type 2 diabetes died more often than those without type 2 diabetes, but the difference was not statistically significant (Table 4).

### Smoking

The day 30 case fatality rate in bacteraemic patients was higher in current or ex-smokers than in nonsmokers (19.7% vs. 1.4%, p < 0.001, RR 16.9; 95% CI 2.1–133.5); ninety-three per cent of patients who died were current or ex-smokers (Table 4). Current or ex-smokers needed ICU treatment (39.4% vs 18.6%, p = 0.007, RR 2.9; 95% CI 1.3–6.2) and mechanical ventilation (21.2% vs 5.7%, p = 0.008, RR 4.4; 95% CI 1.4–14.3) more often than nonsmokers during the bacteremia episode. Current or ex-smokers died more often in ICU treatment compared to nonsmokers (10/26 vs 1/13, p = 0.044, RR 7.5; 95% CI 0.8–66.9) and had high SOFA scores (value > 4 on day 1–3 after positive blood culture finding) more often than nonsmokers (33.3% vs 16.4%, p = 0.029, RR 2.6; 95% CI 1.1–6.0). The current or ex-smoker patient groups did not differ statistically significantly from the nonsmoker group in the occurrence of hypotension (42.4% vs 28.6%, p = 0.091, RR 1.8; 95% CI 0.9–3.8) or in numbers with neurological deficit (lowered GCS) (43.9% vs 32.9%, p = 0.184, RR 1.6; 95% CI 0.8–3.2).

Thirty-eight patients (35.2%) were current smokers while 70 (64.8%) had never smoked. When current smokers were compared to nonsmokers (ex-smokers excluded from this analysis) the adverse effect of smoking for prognosis of bacteraemia was emphasized. The day 30 case fatality rate in bacteraemic patients were higher in current smokers than in nonsmokers (21.1% vs. 1.4%, p < 0.001, RR 18.4; 95% CI 2.2–153.7). Current smokers needed ICU treatment (50.0% vs 18.6%, p = 0.001, RR 4.4; 95% CI 1.8–10.5) and mechanical ventilation (31.6% vs 5.7%, p < 0.001, RR 7.6; 95% CI 2.3–25.8) more often than nonsmokers during the bacteremia episode. This adverse effect remained even after smoking cessation; 5/28 (17.9%) patients died in the ex-smoker group compared to 1/70 (1.4%) of those who had never smoked (p = 0.007, RR 15.0; 95% CI 1.7–135.1). Fifty-nine per cent of males were current or ex-smokers as against 36.9% of females (p = 0.01).

### Alcohol abuse

The day 30 case fatality rate in bacteraemic patients was higher in alcohol abusers compared to those not given to alcohol abuse (29.2% vs 9.6%, p = 0.008, RR 3.9; 95% CI 1.3–11.2). Alcohol abusers needed ICU treatment (66.7% vs 24.8%, p < 0.001, RR 6.1; 95% CI 2.4–15.5) and mechanical ventilation (41.7% vs 9.6%, p < 0.001, RR 6.7;95% CI 2.5–18.4) more often than those not abusing. Alcohol abusers died more often in ICU treatment, the difference being, however, not statistically significant (7/16 vs 8/31, p = 0.211, RR 2.2, 95% CI 0.6–8.0). Alcohol abusers had high SOFA scores (value >4 on day 1–3 after positive blood culture finding) more often than those without alcohol abuse (65.2% vs 21.4%, p < 0.001, RR 6.9; 95% CI 2.6–18.1). The occurrence of hypotension was more common among abusers than in their counterparts (70.8% vs 31.2%, p < 0.001, RR 5.4; 95% CI 2.1–14.0) and were more likely to develop neurological deficit (lowered GSC) (75.0% vs 33.6%, p < 0.001, RR 5.9; 95% CI 2.2–16.0). Eighteen out of 21 (85.7%) alcohol abusers were current smokers or ex-smokers and 4 out of 24 (16.7%) had liver cirrhosis.

The effect of obesity, smoking and alcohol abuse on day 30 case fatality were studied together with age, sex and organism in a multivariate model. Obesity remained a significant risk factor associated with case fatality also in this adjusted model (p = 0.03, RR 6.4; 95% CI 1.2–34.4), together with smoking (P = 0.02, RR 23.0; 95% CI 1.7–321.6).

## Discussion

Obesity, smoking, alcohol abuse, COPD and rheumatoid arthritis proved to be significantly associated with case fatality in bacteraemia in univariate model. The effect of obesity and smoking on case fatality also remained significant when studied in a multivariate model together with alcohol abuse, age (continuos variable), sex and causative organism.

Instead of focusing on the clinical findings associated with poor prognosis in bacteraemia, which have been well studied (such as hypotension, leukopenia or leukocytosis or the number of evolving organ dysfunctions) [17,18], we sought to focus on the underlying conditions and chronic illnesses possibly constituting risk factors for case fatality in bacteraemic patients. Most studies in this field deal with patients with severe bacteraemia requiring ICU treatment [3,18]. One of the major advantages of our study was the enrolment of patients evincing different disease severity; patients with milder symptoms and signs as well as patients with septic shock who needed ICU treatment. One of the limitations was that we could not enrol all patients with bacteraemia in our university hospital district during the study period. This limitation excludes determination of population-based incidence rates. However, the investigators did not select the patients they included in the study, the inclusion being based on the microbiological culture finding, and all patients having the same possibility to be recruited by the investigators from Mondays to Thursdays during the study period. Since the clinicians were unable to know any details about the patients nor their disease severity before the recruitment, the selection was purely based on the blood culture finding. This kind of recruitment of patients probably did not cause any selection bias.

Obesity emerged as an independent risk factor for case fatality and obese patients died more often despite ICU treatment compared to nonobese patients. The published studies examining the association between obesity and in-hospital case fatality give conflicting results concerning the role of body mass index (BMI) as a risk factor for in-hospital case fatality. We found no studies reporting increased mortality due to bacteraemia among obese patients. There are three papers reporting an increased obesity-related case fatality rate in ICU [19-21], these involving patients with multiple reasons for ICU admission, not only infectious causes. In contrast to our study there are also studies where high BMI is not found to be a predictor of case fatality in ICU patients [22-25], and where a low BMI is independently associated with higher case fatality [22,23].

The physiologic mechanisms prevailing between obesity and mortality are unknown. It remains obscure which factors contribute to the increased case fatality in obese in-hospital patients reported in some series. One study showed that obesity exacerbates sepsis-induced inflammation and microvascular dysfunction in the mouse brain [26]. The investigators in question noted microvascular inflammatory and thrombogenic responses, including activation of endothelial cells with subsequent expression of adhesion molecules such as P-selectin in obese mice [26]. Bornstein and associates found that plasma leptin levels are increased in survivors of acute sepsis [27]. The group found that mean leptin levels were three-fold higher in patients who survived the episode than in non-survivors, and concluded that in addition to its function as an anti-obesity factor, leptin may play a role in a severe stress state such as acute sepsis [27]. Recent findings indicate that obesity is an independent risk factor for lipid peroxidation [28] and impaired endothelial cell function [29]. In addition, there appears to be a chronic low-grade inflammation state with elevated acute-phase mediators, cytokines and soluble adhesion molecules which persists in obese individuals [30,31].

Smoking was an independent risk factor for case fatality due to bacteraemia in our study. The effect was most distinct when current smokers were compared to nonsmokers, but it also remained significant after smoking cessation. Smokers more often needed mechanical ventilation and ICU stay, their SOFA score was more often higher than in nonsmokers and they died more often despite ICU treatment. Although the importance of smoking cessation has been emphasized in the therapeutic plan of patients with serious infections [32], there are only a few other studies of the effect of smoking on case fatality in bacteraemia. Arvanitidou and colleagues studied epidemiological and clinical features as potential prognostic factors for outcomes of hospital-acquired bacteraemia in a tertiary care teaching hospital in Greece [14]. They found no differences between non-survivors and survivors in sex, age or smoking habit [14]. Pittet and associates concluded that smoking or alcohol abuse did not reach statistical significance as independent risk factors for case fatality in septicaemia. However, the number of co-morbidities, also including smoking and alcohol abuse, predicted mortality [2].

Alcoholism emerged as a risk factor for case fatality in univariate analysis, this effect being however diminished when studied in an adjusted model together with obesity, smoking, sex, age and organism. This might be explained by the fact that most alcohol abusers were also smokers, and the effect on case fatality may thus result from smoking, not alcohol itself. In accordance with our findings, there are studies showing that alcoholism is a risk factor for case fatality in pneumococcal bacteraemia in univariate analysis [11,12]. There are however also studies where such an association is not confirmed. Lääveri and associates found no statistically significant association between alcohol abuse and increased case fatality in bacteraemic pneumococcal disease [13], while a group under Laupland et al conducted a population-based surveillance cohort study of severe bloodstream infections and found that alcoholism increased the case fatality rate in bacteraemic patients althought the difference was not statistically significant [3].

We had seven patients with rheumatoid arthritis, three of whom died of bacteraemia. The number of patients with rheumatoid arthritis is small, but the results in univariate model suggest that rheumatoid patients have increased case fatality in bacteraemia. Sihvonen and associates conducted a cross-sectional cohort study of rheumatoid arthritis patients and concluded that they carried an increased risk of death from various causes, and they were at increased risk of dying of infections when compared to the general population or controls [33].

## Conclusion

In conclusion, our results indicate that obesity, together with smoking, constitutes an important risk factor for case fatality in bacteraemic patients. With the rising prevalence of obesity in Western countries, future research should focus on finding mechanisms responsible for increased mortality in obese bacteraemic patients. The adverse effect of smoking on bacteraemia outcome is an underestimated health risk. Identification of risk factors underlying fatal outcome in bacteraemia may allow targeting of preventive efforts to individuals likely to derive greatest potential benefit.

## Competing interests

The author(s) declare that they have no competing interests.

## Authors' contributions

All authors planned and carried out the conception and design of the study. J.La., J.S. and R.H. were involved in patient care, and acquisition of data. R.V. was responsible for the blood culture interpretation. All authors were responsible for interpretation of the data. R.H. wrote the first draft ot the manuscript, and all authors participated in its revision. All authors had intellectual contribution, and all read and approved the final manuscript.

## Pre-publication history

The pre-publication history for this paper can be accessed here:


